# Implementation and acceptance of government-sponsored malaria control interventions in Meghalaya, India

**DOI:** 10.1186/s12936-022-04223-5

**Published:** 2022-06-23

**Authors:** Mattimi Passah, Carinthia Balabet Nengnong, Mark L. Wilson, Jane M. Carlton, Larry Kharbamon, Sandra Albert

**Affiliations:** 1 Indian Institute of Public Health Shillong, Shillong, Meghalaya 793001 India; 2 Martin Luther Christian University, Shillong, Meghalaya 793006 India; 3 Department of Epidemiology, School of Public Health, University of Michigan, Ann Arbor, MI 48109 USA; 4 Center for Genomics and Systems Biology, Department of Biology, New York University, New York, NY 10003 USA; 5 Department of Epidemiology, College of Global Public Health, New York University, New York, NY 10012 USA; 6 Department of Health, National Vector Borne Disease Control Programme, Shillong, Meghalaya India

**Keywords:** Implementation evaluation, Malaria prevention, Indoor residual spraying, Long-lasting insecticidal nets, risk perception, Qualitative research

## Abstract

**Background:**

India has made considerable progress in malaria reduction over the past two decades, with government-sponsored indoor residual spraying (IRS) and insecticide-treated bed net (ITN) or long-lasting insecticidal nets (LLIN) distribution being the main vector-related prevention efforts. Few investigations have used non-participant observational methods to assess malaria control measures while they were being implemented, nor documented people’s perceptions and acceptance of IRS or LLINs in India, and none have done so in the northeast region. This study evaluated household (HH)-level operation of IRS and distribution of LLINs by India’s National Vector Borne Disease Control Programme (NVBDCP) in 50 villages of Meghalaya state, and documented their acceptance and use.

**Methods:**

Study field teams accompanied the government health system teams during August-October, 2019 and 2020 to observe deployment of LLINs, and record HH-level data on LLIN numbers and use. In addition, NVBDCP spray teams were followed during 2019–2021 to observe IRS preparation and administration. HH members were interviewed to better understand reasons for acceptance or refusal of spraying.

**Results:**

A total of 8386 LLINs were distributed to 2727 HHs in 24 villages from five Primary Health Centres, representing 99.5% of planned coverage. Interviews with 80 HH residents indicated that they appreciated the LLIN dissemination programme, and generally made regular and appropriate use of LLINs, except during overnight travel or when working in agricultural fields. However, HH-level IRS application, which was observed at 632 HHs, did not always follow standard insecticide preparation and safety protocols. Of 1,079 occupied HHs visited by the spray team, 632 (58.6%) refused to allow any spraying. Only 198 (18.4%) HHs agreed to be sprayed, comprising 152 (14.1%) that were only partly sprayed, and 46 (4.3%) that were fully sprayed. Reasons for refusal included: inadequate time to rearrange HH items, young children were present, annoying smell, staining of walls, and threat to bee-keeping or Eri silk moth cultivation.

**Conclusions:**

These findings are among the first in India that independently evaluate people's perceptions and acceptance of ongoing government-sponsored IRS and LLIN programmes for malaria prevention. They represent important insights for achieving India's goal of malaria elimination by 2030.

## Background

India has made considerable progress in reducing its malaria burden over the past two decades, from about 20 million cases in 2000 to about 5.6 million in 2019 estimated cases, as per the 2020 World Malaria Report [1]**.** Despite the generally declining incidence, malaria remains an important health burden in parts of India, accounting for 88% of malaria cases and 86% of malaria deaths in the World Health Organization (WHO) South-East Asia Region in 2019 [1, 2]. Malaria incidence in India is particularly high in some of the rural regions of the country that are predominantly home to tribal (indigenous) communities [3]. Historically, the regions of India with higher malaria incidence have been located in the east and northeast parts of the country [2, 4].

Primary malaria prevention efforts in India involve two key interventions that can reduce transmission: indoor residual spraying (IRS) and long-lasting insecticidal nets (LLINs). Government operation of IRS and distribution of LLINs in India is performed by the National Vector Borne Disease Control Programme (NVBDCP), whose administrative units are based at the state level within the Government Health Department. IRS is applied to the interior walls of houses in order to repel and reduce longevity of indoor feeding and resting *Anopheles* mosquitoes [5, 6]. LLINs protect individuals who sleep under them by providing a physical barrier in addition to repellent/insecticide that reduces mosquito blood-feeding on humans and mosquito reproduction [7]. Both methods are cost-effective, with LLINs usually lasting for 3–5 years and the residual effect of IRS persisting for about 10–12 weeks [8]. In 2016, as part of an India-wide LLIN distribution programme, over 941,000 LLINs were distributed by the NVBDCP [2] at no cost to households (HHs) in the state of Meghalaya for the first time. The State Programme Officer of the NVBDCP reported that a total of 1,096,077 LLINs were distributed state-wide for a second time in 2020.

A systematic literature review of bed net use to reduce malaria in India demonstrated that people who used either insecticide-treated nets (ITNs) or untreated nets had lower risk than those who did not use bed nets [5]. Another review that quantified the benefits of IRS and compared IRS and ITNs (LLINs) for their ability to prevent malaria in India, concluded that ITNs provided better protection against malaria compared to IRS [9]. A recent study in one block of Madhya Pradesh state, India that monitored the IRS and LLIN campaigns there [10], showed improved LLIN usage and IRS intervention as a result of supportive supervision and monitoring. Surprisingly, few studies have specifically evaluated malaria control measures in India, particularly in the northeast region. An extensive literature search of published reports found no studies on acceptability of LLINs and/or IRS in northeast India. One study in Assam state evaluated the residual bio-efficacy and durability of LLINs [11]. The only other published report, undertaken in Orissa nearly two decades ago as re-treatable ITNs were first being introduced to India [12], is of limited value in understanding India's current malaria prevention efforts.

To understand how contemporary LLIN and IRS interventions are implemented and accepted in practice, this study evaluated through direct observation the household-level distribution of LLINs and IRS by the NVBDCP in 50 villages of Meghalaya, and the acceptance and use by individuals of these two malaria control measures were recorded. The project described here represents the first independent evaluation of the malaria control programmes in Meghalaya.

## Methods

### Study setting

**Fig. 1:**
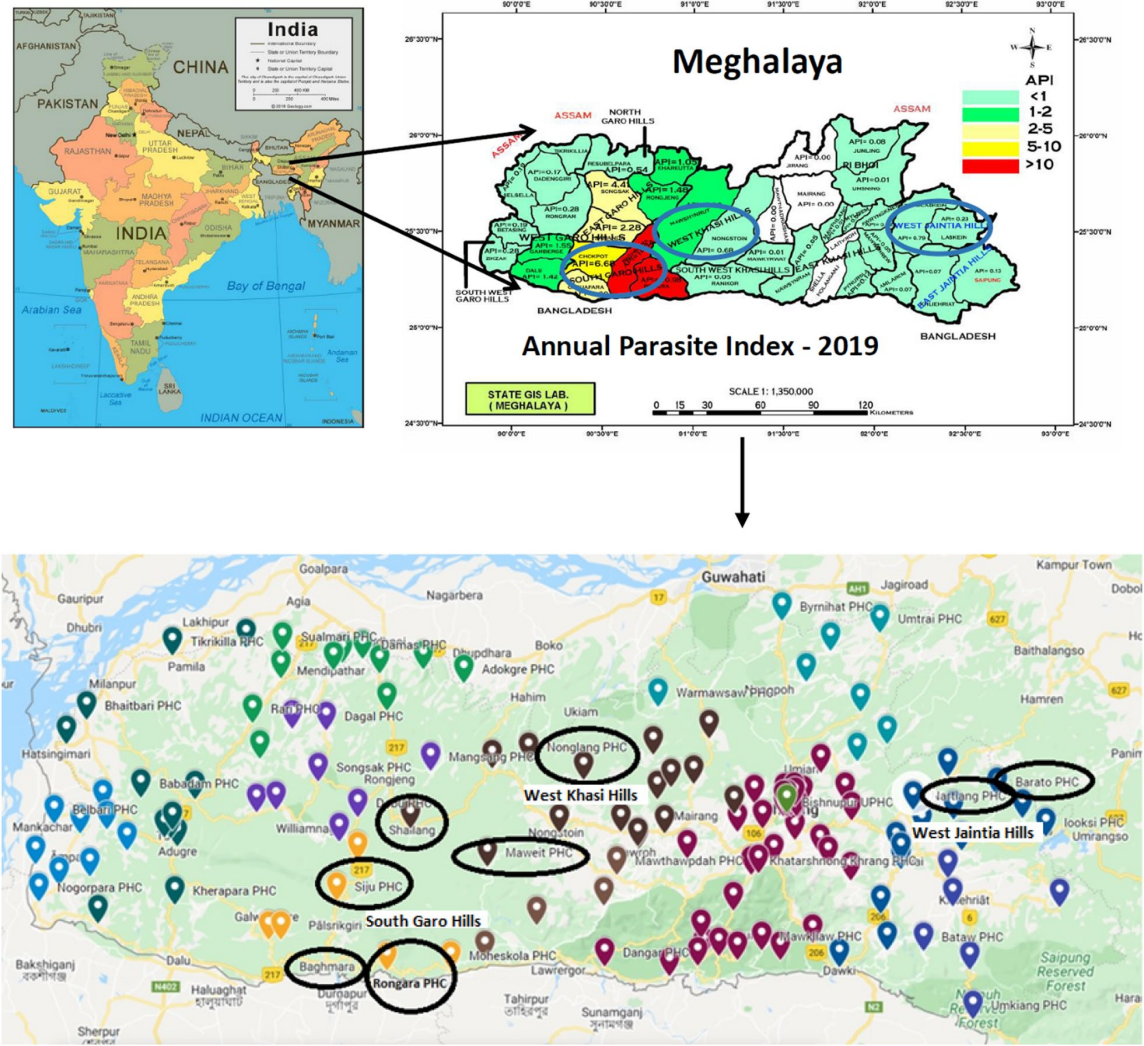
Location of Meghalaya and the study sites. The pins in the detailed map present the PHCs. Each colour represents the PHCs in the different districts. The PHCs selected for the study are circled

Meghalaya is one of seven states located in northeastern India, a region that has borders with Bangladesh, Bhutan, Myanmar, Nepal and Tibet (Fig. 1). An estimated 3.44 million people live in Meghalaya (2021), of which 86% belong to Khasi-Jaintia and Garo tribes that are considered "Schedule Tribes" by the Government of India. Historically, the state of Meghalaya has reported more than 20% of malaria cases in the northeast region of ~ 45 million inhabitants [13], most of which has occurred in the districts of West Khasi Hills (WKH), West Jaintia Hills (WJH), and South Garo Hills (SGH) [2]. The present study was undertaken in these three districts, which also had Meghalaya's highest Annual Parasite Index (API) during 2016–2019 [2]. Data were gathered from a sample of 50 villages served by eight Primary Health Centres (PHCs) in the three districts (Table 1). The data on LLINs pertain to the second round of distribution by the NVBDCP. Due to Covid-19 movement restrictions, IRS observations in two villages of the Nartiang PHC and five villages of Nonglang PHC could not be undertaken. Data were collected from direct non-participant observations, checklists, field notes, and informal interviews.

**Table 1:** Number of villages observed for LLIN distribution, IRS application and LLIN use in a sub-set of Primary Health Centres of three Districts in Meghalaya during 2019–2021

District	PHCs	LLIN distribution	IRS application	LLIN use
West Jaintia Hills (WJH)	Nartiang	6	6	2
	Barato	8	0	2
West Khasi Hills (WKH)	Nonglang	6	1*	5
	Shallang	0	1	0
	Maweit	0	1	0
South Garo Hills (SGH)	Baghmara	3	2	0
	Rongara	1	3	0
	Siju	0	4**	4
Total	8 PHCs	24	18	13

Total adds to 55 villages here as in 1* village in WKH all three activities were assessed. And in 4** villages in SGH two activities were assessed

### Data collection

The study was conducted during September and October 2019 and from August to October 2020. Additional observations were made in the Siju PHC area of the Garo Hills during April 2021. Six trained research assistants (henceforth "field team") worked in pairs to record data on all field observations. Data were initially collected on paper, and then entered, cleaned, and summarized using Microsoft Word and Excel. Field observation and notes were transcribed into Microsoft Word for interpretation.

### LLIN distribution and utilization data

The field team members accompanied the government NVBDCP workers to 24 villages across three districts, to observe and document the LLIN deployment and distribution process (Table 1). During LLIN distribution, the following information was recorded for each HH recipient: reported number of adult and child HH members, number of LLINs allotted per HH (NVBDCP pre-calculated count), number of LLINs actually given to the HH representative, and size of each distributed LLIN. In addition, LLIN programme data were also collected from the responsible State and District officials, including data for those villages where the field team could not accompany the NVBDCP workers for direct observations.

To understand LLIN use at the household-level, a total of 80 households were also visited. HHs were selected using a quasi-random convenient sampling design to represent the different villages. In WKH district, the visits were completed before the second round of state-wide LLIN distribution in 2020, allowing for some before and after comparisons. A brief questionnaire consisting of seven questions was administered to a HH resident (usually the HH head) on the availability and use of LLINs, including the number of LLINs present in the HH, members sleeping under LLINs, reasons for not using LLINs, and other places where LLINs are used by family members.

### IRS data

To observe the spraying process and evaluate HH acceptance of IRS, two research team members accompanied the NVBDCP IRS team (henceforth _"spray team") to 18 villages during the administration of IRS. Selection of households was as per the government protocol where IRS was carried out in PHCs with API > 2. Recorded information included: NVBDCP spray team composition (number and role), spray team interaction(s) with households, method of solution preparation, spraying technique and sites sprayed, number of rooms in HH, number of rooms sprayed, and whether a wall marking that denotes spray status of HH was completed by the spray team.

In a subset of 324 households, a brief informal discussion was held with an adult HH member who verbally consented to participate and was present during the IRS application to gather information on reasons for refusal or acceptance of the spraying. These informal interactions occurred after the NVBDCP administered the IRS. In addition, five of the 30 NVBDCP IRS spray teams were selected, and one member from each of the six teams was also interviewed at the close of day. The first person from each team who consented was interviewed with questions about the roles and responsibilities of the IRS team, the training they had received, and their overall experiences and challenges faced when engaging with the communities.

### Ethical approval

Ethical approval for the study was obtained from the University Research Ethics Committee (UREC) at Martin Luther Christian University, Shillong and Institutional Review Board (IRB) at New York University, New York, NY, USA. Observational field data collection was initiated after obtaining permission from relevant local authorities, such as the village headmen, for interactions at the village level and the Health Department authorities for NVBDCP Health system related activities. Prior to undertaking any observations or informal interviews, the purpose of the research was explained to the NVBDCP team or to HH members, and informed verbal consent was obtained.

## Results

### LLIN distribution and use

#### Village-level observations on LLIN distribution

Distribution of LLINs for each village occurred at a central location, usually a community hall, a school, a sub-centre of the PHC, or the PHC that served the corresponding study village. The NVBDCP guidelines for LLIN distribution specify that HHs should be given one LLIN for an average of 2.5 persons (2 adults or 3 children or 1 adult plus 1–2 children) [14]. The NVBDCP team initially calculated the number of LLINs needed for each village by dividing the village population by 1.8. This was done so that approximately one LLIN was given to every two members of a household. The LLINs that were distributed (Duranet®) came in three sizes (Size 1- 180 × 100x150cm, Size 2- 180 × 130x150cm, and Size 3- 180 × 160x150cm), and the number and size of bed nets actually distributed to each household depended on the number of adults and children present. Almost all households of the study villages received at least one LLIN during the distribution (Table 2).

**Table 2:** Number of LLINs allotted and delivered in 24 selected villages of three Primary Health Centres (PHCs) in three districts of Meghalaya state during June-October, 2020

District	PHC	Village	No. HHs	No. People	No. LLINs Allotted	No. LLINs Delivered
West Jaintia Hills	Nartiang	Mookbu	214	1154	623	623 (100%)
		Bambthong	193	1090	618	618 (100%)
		Latymphu	175	881	458	458 (100%)
		Moobandu	56	280	143	143 (100%)
		Moorathud	80	445	231	231 (100%)
		Nongdhar	99	578	292	292 (100%)
	Barato	Iongkwang	52	546	216	216 (100%)
		Khliehsniriang	87	351	130	130 (100%)
		Mukroh B	158	915	548	548 (100%)
		Mukroh C	155	1016	448	448 (100%)
		Samatan	111	804	433	433 (100%)
		Bhain	32	208	99	99 (100%)
		Thangrain A	233	1579	736	736 (100%)
		Thangrain B	257	1615	771	771 (100%)
West Khasi Hills	Nonglang	Nonglang	238	1574	740	740 (100%)
		Langja	99	390	326	326 (100%)
		Umbyrsit	15	117	61	61 (100%)
		Kyrdum	110	739	320	320 (100%)
		Umwahlang	89	576	336	336 (100%)
		Mawsikar	80	547	259	259 (100%)
South Garo Hills	Rongara	Kosigre	18	91	63	63 (100%)
	Baghmara	Rongdotchi	49	261	153	143 (93.4%)
		Rasnagre	61	356	227	200 (88.1%)
		Domdama	66	417	195	192 (98.4%)
Total	5 PHCs	24 Villages	2727	16530	8426	8386 (99.5%)

In West Jaintia Hills, the NVBDCP staff was observed to be organized and coordinated, having divided themselves into two or three groups that simultaneously covered two to three villages, working from each village's distribution point. NVBDCP staff explained to community members as a group about the proper use and care of LLINs. COVID-19 safety protocols were followed and there was cooperation by the community members. The headmen and a village-based frontline health worker, who is referred to as the Accredited Social Health Activist (ASHA), were present during the distribution process. Additionally, coordination between the local authorities, ASHAs, and PHC staff appeared to be functioning well in delegating the work that was occurring simultaneously at different villages. The field team observed that for the few HH members who could not be present to receive the LLINs, the ASHA, headmen, or neighbours collected nets on their behalf. Overall, informal interactions with the community members in West Jaintia Hills indicated that they were very happy to receive the LLINs, and many said that it was a *‘blessing from the Government*’ (direct quote from study participant).

In West Khasi Hills District, LLIN distribution occurred at the village level, but less uniformly. In Umwahlang and Mawsikar villages, which cannot be reached by automobile, LLIN distribution occurred in a nearby village, Kyrdum. The field team observed understaffing during the distribution process, however the Medical Officer and staff from nearby PHCs often assisted in the distribution. The Medical Officer explained to recipients how to correctly use, maintain and wash LLINs before they were distributed to the community members. Initial miscommunication among new staff was usually resolved by the second day of distribution.

In South Garo Hills District, LLIN distribution was carried out at the village level by a non-governmental organization (BAKDIL) and arranged at the community hall or school. Prior to distribution, an inaugural programme was conducted in the presence of a representative of the NVBDCP team who along with the ASHA and the Field Supervisor of BAKDIL explained the importance of using bed nets, maintaining and washing bed nets, and how to dispose of the water after washing. Instruction on proper disposal of packaging, including that it should not be re-used, was emphasized.

#### HH-level observations on LLIN utilization

Overall, 80 HHs (20 from each of the four PHC areas), representing 449 HH members, were studied for bed net availability and use (Table 3). In West Khasi Hills pre-2020 bed net distribution observations, 12 of 20 (60%) HHs reported owning at least two bed nets, of which 10 HHs reported using untreated nets that they had purchased from the market, and two HHs reported using treated nets. In the 20 HHs observed, 56 of 90 (62.2%) HH members reported sleeping under bed nets (Table 3). Children and mothers were identified as the primary users of bed nets in 16 (80%) of HHs. All 20 HHs reported that they did not use bed nets when they went into the fields.

**Table 3:** Household-level bed net utilization, before and after the 2020 distribution of LLINs

	Pre-distribution (WKH)	Post-distribution (WJH)		Post-distribution (SGH)
Primary Health Centre (PHC)	Nonglang	Nartiang	Barato	Siju
No. HHs sampled	20	20	20	20
No. HH members	90	127	133	99
No. Bed nets available	40	71	48	75
Avg. No. Bed nets per HH member	0.44	0.56	0.36	0.74
HH members using bed nets No. (%)	56 (62.2)	123 (96.8)	116 (87.2)	99 (100)
Bed nets used away from HH	NA	13 (65)	9 (45)	8 (40)
No. HH reporting not staying overnight in fields	NA	7	11	4

Pre-distribution observations occurred in Nonglang (West Khasi Hills), and post-distribution observations were made in villages under Nartiang and Barato (West Jaintia Hills) and Siju (South Garo Hills) PHC

Of the 40 HHs in West Jaintia Hills and 20 HH in South Garo Hills observed following LLIN distribution, 96.8% of members in Nartiang, 87.2% of members in Barato, and 100% of members in Siju reported LLIN use (Table 3). While all HHs reported that at least one HH member was using the new LLIN(s), a few households acknowledged that they continued to use older, untreated bed nets already in their possession. All family members reported sleeping under bed nets in most of the HHs, but in a few instances partial use of bed nets due to insufficient numbers continued to be reported. In West Jaintia Hills, 13 (65%) HHs from Nartiang PHC and 9 (45%) HHs from Barato PHC reported carrying the new LLINs to agricultural fields (Table 3). In South Garo Hills, eight of 20 (40%) HHs reported using bed nets in agricultural fields, six reported not having extra bed nets to take to their fields, four families did not stay overnight in fields, and two families shared that they do not use bed nets in the fields but would if they were available.

### IRS application and HH-level perceptions

#### IRS preparation, interactions and administration by spray team

Each NVBDCP spray team used a standardized protocol (“action plan”) for indoor spraying of DDT that began with a pre-determined subset of HH visits in the villages selected for IRS intervention. The field team observed the spray team while visiting a total of 1,079 HHs: 359 houses during September–October 2019, 574 houses from August-October 2020, and 146 houses in April 2021. Each spray team squad was comprised of five persons with defined roles: one leader interacted with the HH resident(s) and was responsible for marking treated HHs, two members prepared the insecticide spray from the raw powder, and two members sprayed the insecticide.

During the pre-Covid-19 period (observations on 26 Sept. and 23–24 Oct. 2019), the recommended personal safety equipment (including apron, gloves, goggles and hat) was used only intermittently by the spray team. During the Covid-19 pandemic, the spray team was observed on average for six days per village in 12 villages. Personal safety equipment was used by all spray team members while mixing the DDT powder and also while spraying the houses. However, by the sixth day, members of only one of six spray teams continued using the safety equipment.

Before spraying began, the team leader introduced himself to each HH representative and explained the advantages of IRS. It was observed that permission was always obtained from the HH owner before spraying. For those HHs that refused IRS (discussed below), the spray team leader would enquire about the reason for refusal. Each HH that was visited by the spray team was marked with white chalk on the exterior house wall or door, regardless of whether or not it was sprayed. If a house was sprayed, the IRS team would note on the wall the HH number, number of rooms sprayed, total number of rooms, date of spraying, and whether it was first or second round of spraying.

Observations of DDT mixing, spray administration, and disposal of excess DDT were made in 18 villages (Table 4). On asking about the dose of DDT to water, the sprayers were aware about the dose of the insecticide i.e., 1 kg per 10 L of water. The spray teams mostly prepared an approximation of the WHO recommended 1 kg DDT per 10 L water (100 g/L) using a mug without a scale in 15/18 villages. Sprayers stated that the mug held 0.5 kg of DDT powder, and usually 2 mugs of powder were added to ~ 10 L of water. But on one occasion in West Khasi Hills, the spray team was observed mixing 2 kg of DDT powder in 8 L of water, representing 250 g/L. The IRS solution was prepared on the HH premises when the residents accepted the treatment.

**Table 4:** Number of households visited for IRS treatment in 18 villages of three districts by the NVBDCP spray team, and number of households that accepted IRS during observations in 2019, 2020 and 2021

District	Village	Total HH in village	No. (%) HHs visited	No. (%) HH locked	No. (%) HH fully sprayed	No. (%) HH partially sprayed	No. (%) HH refused spraying
West Jaintia Hills	Iongshiwiat	58	55 (94.8)	12 (21.8)	0 (0)	13 (23.6)	30 (54.5)
	Mookhangkhla	113	100 (88.5)	36 (36.0)	1 (1)	5 (5)	58 (58)
	Bamkamar	267	204 (76.4)	87 (42.6)	2 (0.9)	12 (5.9)	103 (50.5)
	Mooker	23	23 (100)	2 (8.7)	1 (4.3)	0 (0)	20 (86.9)
	Lumkya	42	39 (92.9)	14 (3.6)	1 (2.6)	1 (2.6)	23 (58.9)
	Laru	31	27 (87.1)	10 (37.0)	2 (7.4)	2 (7.4)	13 (48.1)
West Khasi Hills	Nonglang	239	133 (55.7)	14 (10.5)	15 (11.3)	10 (7.5)	94 (70.7)
	Nongdhar	33	15 (45.5)	3 (20.0)	4 (26.6)	7 (46.7)	1 (6.6)
	Shallang Kyllonmathei	87	11 (12.6)	0	3 (3.4)	1 (1.1)	7 (8.0)
South Garo Hills	Arapara	110	110 (100)	8 (7.3)	8 (7.3)	0 (0)	94 (85.4)
	Dabram	121	116 (95.9)	8 (7.0)	5 (4.3)	4 (3.4)	99 (85.3)
	Amongre	29	29 (100)	6 (20.7)	1 (3.4)	3 (10.3)	19 (65.5)
	Inolgre	40	40 (100)	2 (5.0)	1 (2.5)	9 (22.5)	28 (70)
	Bolgisigittim	31	31 (100)	5 (16.1)	1 (3.2)	2 (6.4)	23 (74.2)
	Garugittim	38	38 (100)	10 (26.3)	0 (0)	9 (23.7)	19 (50)
	Rongsu Agal	53	53 (100)	12 (22.6)	0 (0)	41 (77.3)	0 (0)
	Rongrigittim	29	29 (100)	5 (17.2)	1 (3.4)	23 (79.3)	0 (0)
	Rongwak	26	26 (100)	16 (61.5)	0 (0)	10 (38.5)	1 (3.8)
Total (%)		1370	1079 (78.7)	250 (23.2)	46 (4.3)	152 (14.1)	632 (58.6)

Although WHO guidelines indicate walls should be sprayed in a downward motion from roof to floor, in 30 of 114 households spraying was performed only under beds and on the ceiling, not on the walls. Proper disposal of the left-over IRS DDT-solution in an appropriate waste pit was not always performed. Instead, the DDT-solution was disposed of in the surrounding environment by the spray members in 14 of 18 villages observed and by all six spray teams. Other inappropriate use of DDT was observed in two WKH villages where some house owners requested and received ‘raw’ DDT powder from spray team members. Villagers reported that they applied the DDT powder to toilets/outdoor, cattle sheds, and as a DDT paste at the bottom of fruit trees. In WJH, the IRS Team indicated that people also asked for ‘raw’ DDT powder, but the requests were not granted.

During informal conversations, some spray operators indicated that they received training before the start of the IRS programme every year. Nevertheless, the field team also observed occasional inappropriate use of DDT. For example, the IRS solution was sprayed outdoors in West Khasi Hills district, with the explanation that the spray teams sometimes adapted their practices at the request of households.

One spray team member in WKH stated that:

*“if we don’t spray outdoor then the villagers won’t allow us to spray indoors, or else none of the houses will accept the spray [IRS]”.*

Sprayers also shared that many HHs in some WKH villages stopped accepting the IRS spray because they feared this affected their rearing of Eri silk worms which contributes to the local economy. Some spray team members also described the challenges faced with the low remuneration for their work, and that the teams have less manpower and a greater work load.

Despite these problems, there appeared to be good coordination among the Senior Malaria Inspector, Surveillance Inspector, Surveillance Worker, Accredited Social Health Activist (ASHA), and the spray team. Furthermore, NVBDCP staff members at the PHC level received regular quantitative summaries from the spray team after they visited each village.

#### Coverage of IRS by spray team and acceptance at HH level

The field team observed NVBDCP spray team interactions with 1,079 households in 18 villages (Table 4). This represented more than three-quarters of the 1,370 occupied houses identified from the ASHA register during 2019, 2020 and 2021. Most of the unvisited houses were in two villages that were not completed (i.e., all HHs were not visited) because the IRS activities were stopped due to the approaching winter season. Of 1,079 observed visits, nearly one-quarter of the houses were locked, with no one apparently home. Thus, only 829 HHs had at least one resident present who could agree to the IRS treatment. Of these, 632, or about three quarters of HHs that responded to the spray team refused to allow any spraying. Thus, the field team observed spraying at 198 HHs, which included 152 (76.8%) houses that had some but not all rooms treated (HH was partially sprayed), and 46 (23.2%) houses in which all rooms were treated (HH was fully sprayed) (Table 4).

#### Reasons for IRS Refusal

An explanation why HH respondents refused to have any rooms in their homes sprayed was provided from 324 dwellings. The primary reason was that no adult was present (29%). Additional reasons for refusal included insufficient time available to rearrange HH items, children under two years old were present, dislike of the smell, not wanting to deface newly painted walls, and potential negative effects on practices, such as bee-keeping and Eri silk moth cultivation (Table 5). Perceived effects from DDT exposure on sensitive groups likes pregnant mothers, unwell family members, and children below two years old were also common responses for refusal. Some HHs believed that IRS had to be done outdoors as well if it was to be beneficial.

**Table 5:** Most important reason for refusing IRS provided by one resident of 324 of 632 houses that would not allow IRS application. Only the first, unprompted reason was considered

Reason for refusal of IRS	WKH (n = 8)	WJH (n = 227)	SGH (n = 89)	Total no. (%) responses
No adult present during the visit	3	67	24	94 (29.0)
No time to rearrange HH items	–	35	8	43 (13.3)
Presence of children < 2 years old	–	30	2	32 (9.9)
Dislikes smell of DDT	2	8	21	31 (9.6)
Stains walls or harms wall paint	–	18	20	38 (11.7)
Effects on livestock or insect rearing	3	11	–	14 (4.3)
Presence of person with illness	–	10	1	11 (3.4)
Never previously done or explained	–	15	1	16 (4.9)
Not needed or useful	–	5	2	7 (2.2)
Other	–	4	1	5 (1.5)
No particular reasons/does not want	–	24	9	33 (10.2)

*“It will be of no use if you only spray it inside the house as the mosquito can still get inside our house though they won’t enter inside our house immediately after the spray but these mosquitoes will try to rest first outside our house either in the veranda or the surrounding of the house and after sometimes they will start entering the house but if you also spray it outdoor there is no chance of the mosquitoes of entering inside our house as there is no place from them to rest within our surrounding areas”.* (Elderly person, Kyllonmathei Village, WKH).

The HHs that accepted IRS often said they believed it was effective in malaria control and that DDT not only kills the mosquitoes but other insects as well. Awareness by the NVDBCP technical team about the advantages of IRS contributed to the community members acceptance of the intervention.

*“The Surveillance Inspector from the PHC has explained to the communities about the importance of IRS. Even during the LLIN distribution we had few months back, when he explained about the LLINs he also spoke about how IRS can help in malaria control in the community”* (ASHA, WKH).

## Discussion

LLINs and IRS are the primary government-administered prevention methods used for malaria control in India. Considerable evidence has shown both of these interventions to be effective at reducing *Plasmodium* transmission and malaria disease [5–7, 15–17]. A systematic review and analysis of 11 years of NVBDCP malaria case data for Meghalaya reported a decrease in cases and malaria-related deaths, which is most likely due to changes in treatment and/or the state-wide distribution of LLINs in 2016. According to a state monthly malaria cases report during the year 2019, of the 869 fever cases tested for malaria only 3 were positive. Kessler et al. assessed the impact of mass LLIN distribution in the state of Meghalaya for the year 2015 (year preceding LLIN distribution), 2016 (year of LLIN distribution), and 2017 (year following LLIN distribution) in the seven malaria-affected districts of the state. That study reports dramatic reduction in malaria cases in four districts, namely West and East Garo Hills, Ri Bhoi, and Jaintia Hills in 2016, which is the same year that the first distribution of LLINs occurred. In West Khasi Hills, the decline in reported malaria occurred mainly in 2017. In South Garo Hills, there was no reduction in reported cases during the years examined [44]. An early study of ITN feasibility and acceptability in India occurred in Orissa state during 2003–2004, when IRS had been withdrawn and ITNs were being introduced by the NVBDCP [12]. That report is historically interesting, but of little relevance today, as it identified people's general unwillingness to purchase ITNs and to pay for their required retreatment, even though ITN use was high if community members perceived a health benefit. The present study revealed that, during a government-sponsored, free net distribution programme, LLINs had been widely distributed to populations at risk, with ownership increasing in HHs that did not own a treated bed net before the programme.

Village-level LLIN delivery generally functioned smoothly and covered ~ 99% of the study HHs. These findings are similar to another observational study conducted in Madhya Pradesh, India where the average coverage of LLINs following a government distribution was also ~ 99% [3]. A Randomized Control Trial in Odisha, India analysed whether micro-loans increased ownership and use of ITNs, and suggested that in the context of interest in using nets, high cost was an impediment to purchasing them. That study indicates people's interest in using ITNs, and the value of government programmes that facilitate easy access [18]. Studies in other nearby countries (e.g. Cambodia, Lao PDR, Myanmar, Vietnam) have shown less success in distributing and encouraging use of LLINs unless information is tailored to the local culture [19–21], or the population is residentially stable [22–24].

Although LLINs were reportedly used frequently at home, this was less often true when people stayed overnight in another village or agricultural fields, especially when people desire to use nets but they are unavailable [25]. Many other studies have shown that ITNs are regularly used in high transmission settings if they are widely available at no cost, and if people understand their value and proper deployment methods [25–27]. An early LLIN study (2003) in Odisha, India, which evaluated participant's willingness to purchase LLINs and their amount of use, more than three-quarters of respondents perceived benefits of using LLINs, and more than half expressed their willingness to buy them [28]. In our study, the vast majority (87%) of participants reported using LLINs while sleeping within their HHs, but only half said they took bed nets to their agricultural fields. Similarly, one-third of people in a Madhya Pradesh, India investigation reported carrying LLINs in 2017 when sleeping outside their homes, but that increased to three-quarters of respondents in 2020 following enhanced explanation of use and value [10]. Another 2009–2010 qualitative study in Odisha [29] reported that few participants reported using mosquito nets regularly, despite being aware of the benefits. Low ITN use reportedly varied according to season, cost, perceived toxicity and alternate net uses.

This study results suggest how improving education and government-provided services could make an improvement in more effective prevention and control. Other studies in Uganda, which investigated associations between overnight travel and risk behaviours in relation to *Plasmodium* infection, evaluated use of LLINs and other personal protection measures by travellers to prevent malaria [30, 31]. Those results suggest that even people with good knowledge of malaria risk make limited use of LLINs during travel, and travellers to locations that had not received IRS were more likely than non-travellers to have a malaria diagnosis [32]. Similar findings were reported among Cambodian youth sleeping away from their houses [24]. Furthermore, a recent investigation in Zambia [33] concluded that ITN use while traveling reduced *Plasmodium* infection risk by 35% over non-users. Other work in Namibia has shown that overnight cross-border travel increased malaria risk tenfold over domestic travel, with night-time outdoor agricultural work doubling the risk [34]. The reasons for not taking or using an ITN while away from home include social, logistical and psychological, with evidence from one study indicating "social disapproval" of disrespect or display of wealth [35]. This study, however, one main reason for not using LLINs during travel was not having extra bed nets to take from home. Education about the importance of carrying and using bed nets when away from home could reduce malaria risk to travellers and agricultural workers.

In the present study, village-level distribution of free LLINs occurred at one focal location (e.g., community hall, school), and was closely accompanied with educating community members on bed net use practices. This may have increased the acceptance and subsequent use of LLINs that was observed. Alternatively, in Zambia [36], it was shown that door-to-door delivery of LLINs ensured availability in hard-to-reach areas, and provided a good opportunity to educate the members face-to-face. Education and awareness activities during and after net distribution also have played a key role in increasing compliance of LLIN usage in other settings [37]. Investigations in nearby Myanmar [38–40], as well as Ethiopia [41] and Kenya [42], for example, show that perceptions of ITNs and their use depend on knowledge and beliefs about malaria risk, as well as appropriate, culturally sensitive education. The extensive MEDP programme in Madhya Pradesh has demonstrated the critical importance of "context-specific IEC" among other logistical and policy considerations [43].

The widespread acceptance and use of LLINs following the India nationwide campaign in 2016 may have been a principal reason for the sharp decline in malaria incidence in Meghalaya in general, and specifically at the sites of this study [2, 44, 45]. This decline in malaria has continued through 2021, and seems in part attributable to the widespread use of LLINs throughout the region. However, as malaria incidence decreases, and other factors lower people's perceived need for malaria protection, ITN use may decline [46–49]. In addition, as LLINs become damaged and loose insecticide potency, they become less effective [11, 50, 51]. Because the present study was cross-sectional, insights into whether villagers continued to use bed nets well after distribution cannot be made. For these reasons, the continued use and effectiveness of LLINs should be regularly monitored to avoid malaria resurgence in the future [52]. Another 2014 study in Odisha, India [53], which assessed the insecticidal efficacy, usage pattern, washing practices and physical integrity of PermaNet LLINs, identified the importance of maintaining LLIN programmes and continuing periodic distribution of new nets to replace those that have holes and have lost insecticide concentration [53]. Despite a recent decline in malaria incidence in this study area, many residents still continued to use LLINs at home, and to a lesser extent while away from home.

IRS coverage, however, was observed to be generally incomplete and much less accepted by people in the study area. Of more than a thousand households visited by the NVBDCP spray team, less than 5% were fully sprayed, with an additional 14% being only partially sprayed. These findings are similar to a cross-sectional study done in the urban settings of the East Khasi Hills district of Meghalaya which reported IRS not being regarded as an effective measure and not accepted by most of the community members due to reasons such as poisoning food items, cause foul odour and stains on the walls [54]. Studies in Jharkhand shows IRS coverage which ranges from 30 to 50% and 67% had lost their faith in DDT spraying as it is ineffective [55, 56]. Indeed, about two-thirds of the HHs refused spraying, and nearly one-quarter were "locked" (no one responded) when approached by the spray team. Acceptance of IRS is often problematic, with advocacy campaigns being geared towards preparing communities for IRS, with the goal of achieving high initial coverage [57]. A few studies have reported IRS acceptance to be generally high, including an investigation in Tanzania [58] where about 95% of participants reported agreeing to receiving IRS, primarily because they recognized that mosquitoes transmitted the malaria parasite and the spray reduced mosquito abundance. Another qualitative study in Namibia that evaluated people's acceptance of case-reactive IRS of households [16], virtually all participants accepted the IRS, in part because they perceived the importance of malaria risk, would receive free healthcare with the IRS, and were educated by respectful study team members. Most studies, however, reported extensive reluctance and strong resistance to household IRS, including reports from Ghana [59], Iran [60], and Mozambique [61], for example. Major reasons commonly offered for not accepting spraying included that the insecticide stained walls, contaminated food, smelled badly, and caused headaches or difficulty breathing. Logistical challenges included difficulty in moving or protecting furniture and other belongings. Lack of information about the IRS programme, perceived low effectiveness of IRS, and a preference of ITNs over IRS was also cited. Notably, the selection and behaviour of spray operators as they interacted with household residents was also found to be a deterrent to acceptance.

In the present study, the preparation and application of the insecticide by spray operators appeared to be inconsistent and possibly inadequate. Mixing of the insecticide solution was often not done as recommended, suggesting need for retraining and supervision of the spray team. Interestingly, observations in the present study differ from those in Madhya Pradesh, India [62], where proper preparation of the spray solution occurred in 99% of observations. and proper mixing and filtering more than 80% of the time. Furthermore, in the present study, proper disposal of the unused IRS DDT solution in an appropriate waste pit was only seen 22.2% of the time. Indeed, disposal of DDT-solution in the open environment was common across all spray teams and in every village. Similar inappropriate insecticide handling and logistical challenges have been reported in other settings [63]. Unlike the study in Madhya Pradesh [62], it was reported that under supervision, after the completion of the spraying, the leftover solution was used again for spraying in nearly one-fifth of the cases and disposed of in a hole in the ground more than four-fifths of the time [62]. This suggests the need for regular retraining of IRS team members, with an emphasis on human safety and environmental protection [62, 64].

Based on discussion with some HH members, villagers’ knowledge about indoor spraying in the present study varied considerably, but most recognized that it was intended to kill malaria vector mosquitoes or reduce their transmission efficiency. Nevertheless, education of HH residents about the effectiveness and safety of IRS might improve uptake. Increased acceptance of IRS has occurred in some settings following community-based programmes to inform residents about the value of IRS [60]. In addition, residents who are taught about IRS safety better understand how to avoid risks of insecticide exposure [65]. However, the primary reasons that people in the present study did not allow treatment of their houses were related to inconvenience of preparing the space, and undesirable stains and smell.

Community education can be complemented with better training of sprayers to be sensitive to local conditions [62]. Local leaders also have a role to play in reassuring people of the safety of IRS [43]. Indeed, exposure risks to the sprayers themselves could be reduced with proper training, as one study in Ethiopia has shown that many workers perceived risk to be low and had limited knowledge of the occupational dangers they faced [66]. In addition, community members need to be educated about the values of IRS, as shown in a Ghana study that regarded education as the main strategy for managing spray refusals, thereby, facilitating the implementation of IRS [59]. Another recent study in Nigeria concluded that IRS acceptability depends on providing households with information on its effectiveness in reducing the cases, and the costs of malaria prevention [67]. As in those studies, Meghalaya IRS efforts would likely benefit by providing community members with information, education, and communications (IEC) by personnel knowledgeable about malaria prevention methods, and by involving community-based volunteers, chiefs, and opinion leaders in disseminating information at the community level that promotes IRS acceptance [59].

A cluster-randomized controlled trial in Ethiopia that evaluated combined IRS and LLIN use and entomological outcomes reported that these two interventions lowered both density and human biting rate of malaria vectors [68]. Use of LLINs and IRS together to control the vectors responsible for outdoor transmission is a major challenge for elimination of residual foci. Human activities and behaviour, combined with outdoor-biting mosquitoes with flexible feeding preferences, are among the reasons why LLIN- and IRS-based vector control interventions may be ineffective [69]. Whether IRS provides value added when LLINs are being widely used is not known, and depends on the local context, including transmission intensity and insecticide resistance patterns [17]. A recent cluster-randomized trial in a highly endemic area of Mozambique suggested that despite high ITN access but emerging pyrethroid resistance, use of pirimiphos-methyl IRS provided significant additional malaria protection for children under five years of age [70]. On the other hand, a cluster-randomized trial in The Gambia, with moderate seasonal transmission, susceptible vectors, but high LLIN coverage, found no difference between study groups in vector density or clinical malaria [71]. Whether it is cost-effective to administer IRS in Meghalaya settings of moderate-to-low transmission, and high LLIN use should be evaluated, especially if people's acceptance is low.

Among the limitations of the present study is the cross-sectional nature of the observations. Unfortunately, no pre- and post-intervention observations in the same villages were made, restricting the inferences that can be made. It is possible that the spray team members changed their normal behaviour because they knew that they were being observed and questioned. HH members who responded to questions may have provided answers that they thought were being sought, or would not embarrass them.

On the other hand, the investigation obtained qualitative and quantitative data from many residents of a large number of villages. Results came from direct observations and participant interviews. While perhaps not representative of all of Meghalaya, insights into IRS and LLIN acceptance and use were developed that provide needed understanding for HH- and community-level malaria prevention policy. Economic and other contextual barriers such as IRS interference with Eri silkworm rearing are important factors to address at a local or sub-national level. This is a first of its kind study that did an evaluation of the Government’s malaria control programme by an independent research team through non-participant observation in northeast India.

The government of India has a plan to eliminate malaria by 2030 [4, 72], though many challenges remain [43, 73]. Guerin et al. have highlighted the challenges, such as India's shared border with Myanmar where *P. falciparum* parasites with reduced susceptibility to artemisinin are becoming predominant, and that allow international human movements from the rest of South East Asia region [4]. Findings from that report are relevant to our study setting in Meghalaya, which shares borders with Assam state and Bangladesh, both with malaria-endemic areas. Among the findings of the present study was the extensive use of LLINs within HHs, but much lower use when travelling. Nevertheless, the LLIN distribution programme was seen to be well organized and successful in meeting coverage targets. LLINs have demonstrated high effectiveness in many settings, and malaria incidence in the study area declined dramatically in the past five years, which largely coincided with the NVBDCP net distributions. This programme appears to have been quite effective and well-received, suggesting that the government continue to invest efforts into LLIN distribution for malaria prevention. Efforts to use IRS within people's houses, however, were fraught with problems, most importantly the reluctance of people to permit insecticide spraying. It would seem that IRS provides little additional malaria prevention given the extensive use of LLINs, but this issue needs further study. In addition, it is unclear whether more intensive or thorough IRS, undertaken as the guidelines recommend, would further enhance or improve acceptance and protection. Perhaps use of IRS combined with LLINs and more awareness programmes on the importance of IRS will be helpful as transmission becomes even less intense, if perceived malaria risk declines, and when the goal of local elimination appears more achievable.

## Data Availability

Not applicable.

## Abbreviations

ASHA: Accredited social health activist;
ITN: Insecticide-treated net;
IRS: Indoor residual spraying;
LLIN: Long-lasting insecticidal net;
NVBDCP: National vector borne disease control programme
PHC: Primary health centre.
HH: Household
